# Upon the Management of the Third Stage of Labor

**Published:** 1875-02-15

**Authors:** H. P. Merriman


THE
### MEDICAL EXAMINER.
A
Semi-Monthly Journal of Medical Sciences.

EDITED BY N. S. DAVIS, M.D., AND F. H. DAVIS, M.D.

No. IV. CHICAGO, FEB. 15, 1875. VOL. XVI.

### Original Communications.

### UPON THE MANAGEMENT OF THE THIRD STAGE OF LABOR.

READ BEFORE THE SOCIETY OF PHYSICIANS AND SURGEONS JAN. 25TH, 1875, BY PROF. H. P. MERRIMAN, M. D.

THERE are so many dangers connected with the puerperal state of women that it becomes us as physicians to examine carefully and see to what extent they may arise from an unsatisfactory completion of some stage of labor, since any trouble is more easily remedied when once its origin is well understood.

The accidents most dreaded after the birth of the child are: Inversion of the uterus (rare); Hæmorrhage; Septicæmia; Inflammation of the uterus, or parts adjacent; Sub-involution, with debilitating discharges; Prolapsus.

*Inversion of the Uterus.*—This affection is generally acknowledged to be due to a preternatural delivery of the placenta. It rarely, I may say never, occurs except in the case of a uterus remaining large and flaccid after the expulsion of the child. In this state Dr. William Hunter used to say it was as easily inverted as the finger of a glove; but when it was contracted it was as hard to invert as a jackboot. In this relaxed condition, pulling at the funis to deliver the placenta would readily produce the difficulty. Sometimes the uterus alone effects the inversion by its own expulsive efforts immediately after the birth of the child. Even then, however, it is due to irregular and unnatural contractions, the fibres at the fundus acting strongly while those at and near the cervix are completely relaxed. Dr. Byford gives an excellent description of these cases.*

*Hæmorrhage.*—Dangerous hæmorrhages may have two sources, only

*Medicine and Surgery of Women.

one of which is from the placental site and due to an incomplete contraction of the uterus; perhaps some remnant of the placenta remaining attached to its walls prevents this contraction (See the interesting case reported by Dr. Heywood Smith in the Transactions of the London Obstetrical Society, 1869). The other form of hæmorrhage occurs from the *cervix uteri* on account of a rupture during the second stage of labor, when the os does not readily relax. This happens more frequently than is generally supposed.

*Septicæmia.*—The views held upon this fearfully fatal affection are so various and so extensive that in such introductory notes as the present, allusion can be made only to those theoretical causes which recognize the disease as originating from the presence of decomposing material in, and its absorption from, the uterus. Careful observers testify to the frequency of a profuse hæmorrhage in those cases of labor which are followed by puerperal fever, thus making a close connection between an abundant material for decomposition with a relaxed state of the uterus on the one hand, and on the other the most terrible puerperal disease. It seems natural to accept the absorption of putrid matter as the cause of this disease.

Then if the third stage of labor is so conducted as to secure a well-contracted uterus, it prevents the accumulation of material for decomposition; the absorption of putrid matter by a large and relaxed placental surface; lastly, the venous system will not be in a state to absorb rapidly, since it will have suffered no large loss of blood to empty the vessels and make them *hungry*. A full state of the blood-vessels is a good means of preventing absorption.

*Inflammations.*— Dr. Gooch says: "There seems rather a particular disposition to inflammation of the uterus after uterine hæmorrhage." It is then easily excited by local injuries, cold, and other causes. All authors regard decomposing material in the uterus as a prolific source of metritis, endometritis, peritonitis, and pelvic inflammations. These often result from the absorption of putrid matter to an insufficient extent for complete septicæmia.

*Sub-involution of the Uterus.*—The discharges which attend this condition of affairs are usually profuse and exceedingly debilitating, and become a fruitful source of aches and pains, producing a chronic state of invalidism.

Since, then, we find the dangers of the puerperal state, dependent to so large a degree upon a complete and satisfactory conclusion of the third stage of labor, we may take as our own an axiom of Dr. Barnes, slightly modified. "By the proper management of the third stage of labor, we greatly secure our patient against hæmorrhage, and many other dangers."

Let us see in what proper management consists. Before determining upon our own course, it will be well, I think, to consider the methods of practice adopted by the great men whose names have illumined our profession both in ancient and modern times.

I commence with the father of modern midwifery. Francis Mauriceau (1668) directs that as soon as the child is born, "before they do so much as tie or cut the navel-string, they must, without losing time, free the Woman from this fleshy Mass," which he says may be done by drawing gently and from side to side on the cord, being careful not to use too much violence "lest by breaking the String near the Burthen, as sometimes happens, you be obliged to put up the whole Hand into the Womb to deliver the Woman; or that the Womb, to which the Burthen is sometimes strongly fastened, be not drawn forth with it, as hath been done to some that I knew. As also in drawing it forth with too much Violence, there may happen a very great Flooding, which would be of dangerous Consequence." He advises the woman also to assist, by blowing into her hands, by holding her breath, and by bearing down as in labor. If all of these are not sufficient, he says, "You may command an experienced Nurse-keeper to press the Belly lightly with the Flat of her Hand, directing it gently downward by way of Friction—above all, being careful not to do it too boisterously. But if all this be in vain, then must the Hand be directed into the Womb to loosen it and separate it."

In speaking of the care of a woman after her confinement, he alludes to the custom of physicians "to direct a small Plaister of Galbanum, with a little Civet in the middle to be apply'd to the Woman's Navel, which, as they imagine, is very proper to keep the Womb in its Place, because, being delighted with that Smell, it draws near to it of itself; but this Remedy is a little superstitious! wherefore I am not for it, it being sufficient to keep the Belly very warm in the situation we have directed, and to prevent the least cold."

I very much admire this old author, among other things for the very decided manner in which he gives up old customs which have nothing to recommend them except that they *are* old. When Hugh Chamberlen translated this work into English, he put in a foot-note to this direction of Mauriceau as follows: "Practice and Success commend it, nor is there Reason wanting to defend it; wherefore, notwithstanding the Author's Sense, it may be successfully continued."

John Burton of York (1756) advises to introduce the hand immediately (in a general way), or as soon as may be after the expulsion of the child, and to carefully remove the placenta. For this advice he gives seven reasons: 1, because the os and vagina are then widely dilated by the recent passage of the child, and there will be little difficulty or pain; 2, you may thus discover another infant; 3, you may thus discover the presence of a mole, polypus, or any other abnormal thing; 4, you may thus discover and remove adhesions; 5, you can remove fragments, clots, etc.; 6, you can prevent the descent and fall (inversion) of the uterus; 7, you can learn the condition of the uterus as to atony, etc., and the presence of your hand will stimulate contraction. In this procedure he says the operator should not follow the navel string, as that would conduct his hand within the membranes and would embarrass his manipulations, but he should introduce his fingers between the membranes and the womb, when he will have little difficulty. He must take care to do it gently, and not to scratch the womb with his nails. He says three objections have been made to this procedure: First, that it gives pain; secondly, that there is a difficulty in finding out the placenta; thirdly, that there is danger of leaving some fragment. The first objection, he says, is already answered, and not one woman in five hundred is ever sensible of the matter because of the greater dilatation that preceded it. To the second objection he replies, "any botch in midwifery can easily distinguish the womb from the placenta." The last objection he reregards as of no weight, for there cannot be more danger of leaving a fragment than when the placenta is tugged out by the funis.

W. Smellie (1766) alludes to the difference of opinion about delivering the placenta, some alleging that it should be delivered slowly, or left to come away of itself, others that the hand should be immediately introduced into the uterus to separate and bring it away, and adds it should be considered how nature herself acts. In his opinion we ought to go in the middle way, never to assist but when we find it necessary; on the one hand not to torture nature when it is self-sufficient, nor delay help too long.

I may abridge my account by perhaps a general summing up of the teachings of the last part of the eighteenth century and of the first half of the present. It has been with few exceptions the plan to advise patient waiting without interference, when there was no hæmorrhage, until the uterus had itself separated and expelled the placenta into the vagina. This teaching compelled Velpeau, as he tells us,* during the space of only six months in 1823, to wait at one time ten hours; at a second twenty-four hours; at a third thirty-six hours; and at a fourth case forty-eight hours, and even then he had to introduce his hand for the removal of the placenta!

Coming now to the practical part of our subject, let us consider for a moment the *mechanism of the expulsion of the placenta.* This has not been studied as generally or as carefully as its importance demands, and authors are far from agreed upon the method which nature herself takes to accomplish this important stage of labor.

Baudelocque and Schultze taught that the central part of the placenta is usually first separated by the uterine contractions, producing a cup-like space that becomes filled with blood, and this accumulation co-operates in pushing off the remaining attachments. Thus we should have the fœtal surface of the mass presenting at the *os uteri* and the membranes would be expelled, inverted, or turned inside out, which Ramsbotham, Rigby, and many other prominent authors, regard as *the* natural method.

A more modern view is that the uterine contractions separate the edges of the placenta first, and then roll them over on themselves, the roll being in the line of the uterine axis. By this process the shape of the mass and the direction of its axis is such as to make the delivery easy. (This is one of the economies we expect of nature, which takes the most ready manner of accomplishing her work.) There would be little hæmorrhage necessary by this method, while considerable *must* occur by the former. This view is held by Lemser,* Caseaux, J. Matthews Duncan,† Leishman,‡ and many others.

*Meigs' Velpeau, 1831.

*Physiological Separation of the Placenta.
†Mechanism of the Expulsion of the Placenta.
‡System of Midwifery.

Barnes* holds a modified view: He says when the placenta is attached to the fundus of the uterus the central portion will be separated first. Burton of York, whose experience in introducing the hand into the uterus was so great, said: "I always found the strongest adhesion to be toward the edge of the placenta." This was when he had to separate adhesions.

I think examination will satisfy any one that when there has been no traction upon the cord, the placenta may come in either of these two ways, *i.e.*, inverted or not inverted. I find them in a majority of cases inverted, but in a large minority they come with the fœtal surface inside where no traction upon the cord has been indulged in.

I am convinced that an unmentioned cause of this inversion, when it does occur, is that the membranes in many cases adhere to the uterine walls even after the placenta is separated and expelled into the vagina. Who has not seen many instances of this, even detaining the placenta quite strongly in the vagina, and necessitating the advised twisting of the membranes into a rope, to prevent rupture in drawing them out? Dr. Byford says he has seen fatal cases of septicæmia resulting from membranes left in the uterus.

Why should we not habitually leave the third stage of labor to nature's own efforts? Hunter, Ruysch, Denman, and *many* European authorities encouraged this, at the same time advising not to leave the patient till it was accomplished; but it was found that hæmorrhage and puerperal fever were largely increased thereby.

The consequences of retention of the placenta are:†

*Obstetric Operations.
†Barnes' "Obstetric Operations."

1. Commonly hæmorrhage and spasmodic pain. 2. Sometimes no hæmorrhage, but expulsion after an indefinite number of hours or days. 3. Decomposition of blood, producing horribly offensive discharges; if the placenta blocks up the orifice, we may have enlargement and tympanitis and the condition called physometra. 4. Septicæmia. 5. Inflammations.

There is a liability to these even where the placenta has been expelled, if large clots are allowed to collect and decompose in the uterus; consequently the third stage of labor is not properly completed until the uterus, besides being thoroughly emptied, is also firmly contracted.

It is necessary then sometimes to interfere, and the questions at once arise: When shall assistance be rendered, and secondly, in what manner will assistance be rendered most invaluable to our patient?

Let us examine nature's method, in her happier instances. Immediately after the birth of the child, there appears to be for some little time (say ten minutes) a cessation of the uterine efforts; then they are resumed in the shape of after pains for the separation and expulsion of the placenta. . . . During this period of cessation of *active* contractions, the uterus needs to be maintained in a *tonic* contractile state, otherwise we shall have considerable hæmorrhage whenever the placenta has been partially detached during the second stage of labor. (Authors generally now bring out the distinction between active contractions and a *tonic contractile state* which firmly holds what has been already gained.) . . . Now if the hand be laid with a firm but gentle, careful grasping pressure upon the fundus of the uterus, as it expels the child, following it down and then continuing it during this time of waiting, it will prevent relaxation and will maintain the uterine fibres in such a tonic state that little bleeding will occur. By this method we are imitating nature, in allowing a rest and yet maintaining the condition already gained. Another advantage we have is that when the uterine efforts are resumed, they are far more efficient than when the uterus has been relaxed for some ten or fifteen minutes, and has become filled with blood in a clotted mass. In ordinary labor this seems all that it is best to do. When the patient has had two or three pains, the decreased and hardened condition of the uterine globe will usually indicate the complete or partial expulsion of the placenta from the cavity of the uterus, and an oiled finger introduced into the vagina will hook it away with little difficulty. There is no objection to drawing upon the cord after the placenta can be felt in the vagina. The same sort of supporting pressure upon the uterus should be continued for at least half an hour longer, to prevent relaxation and hæmorrhage. When the uterus has remained firmly contracted for that length of time in a *healthy woman*, she may be regarded reasonably safe.

Cases requiring more decided interference may be grouped under three heads. Where the placenta is retained from—

1. Atony of the uterus.
2. Irregular contractions (rare), and
3. Morbid adhesions (very rare).

Physicians do not require a description of the symptoms of atony; it is readily recognized. It rarely occurs, except in debilitated women, or after a protracted and debilitating labor. The cause of atony I believe to be a temporary exhaustion of nerve force from excessive use; and I think we should have far less trouble from it in either the second or third stage of labor, were we to give our patient, on its earliest symptoms, three or four grains of quinia. This is like giving a tired horse a peck of oats, while the other remedies that merely stimulate the uterus are like a whip, which is sometimes very useful in enabling us to get increased work from a vigorous animal, but which fails when the animal is exhausted beyond measure. *We must treat the patient as well as the uterus.* A reason for the complete failure of uterine stimulants in so many instances has been the utter exhaustion of the woman when they were applied.

One of the methods of stimulating the uterus is to give ergot; another is to apply external stimulation—pressure, cold and electricity.

There is an objection to the use of ergot for the expulsion of the placenta, as not infrequently it produces irregular contractions of the uterus, which retain, rather than expel, the placenta. The other class of stimulants to the uterine fibres will be found sufficient, and are always preferable as far as delivery of the placenta is concerned, though for atony continuing after that event the ergot is certainly advisable, in connection with tonics. All the effects of ergot cannot be discussed in an article like the present.

As to the methods of using compression. For generations English physicians have been taught to lay a hand upon the fundus of the uterus, and press it strongly down into the pelvis, thus securing uterine contraction. Crede's method, as I understand it, differs but little; it is to grasp the uterus strongly, almost immediately after the second stage of labor is completed, and then to squeeze out the placenta and clots as one empties a grape of its contents. After it has contracted strongly, the decreased size will indicate when the placenta is expelled into the vagina. When, in rare instances, the uterus does not act under this stimulus, dangerous hæmorrhage may occur. Under these circumstances the physician must introduce his hand into the uterus, and with the utmost care detach the placenta, but leave the expulsion of his hand enclosing the mass to uterine contraction, which will be strongly stimulated by this contact. This manœuvre must be skillfully executed, the right hand well oiled being insinuated in the gentlest manner possible, while the left steadies and supports the fundus through the abdominal walls.

We shall not need to use *cold* or *electricity* for the delivery of the placenta, but the hæmorrhage sometimes occurring afterward may require them, and they are then very efficient.

Of these three methods of applying external stimulation, that of Crede is the readiest, and for that reason will be oftenest brought into requisition; cold stands next in convenience of application, and should be applied in such a manner as to cause shock and thus arouse the nervous energies: *e.g.*, by laying a cold hand suddenly upon the abdomen, by slapping it with a wet towel, or by pouring cold water upon it in a steady stream from some little height. Electricity often is not available when needed, but when at hand is one of the most effective means we possess of securing uterine contractions. (See reports of Drs. Earle and Danforth, in MEDICAL EXAMINER, Feb. 1st, 1874.) The faradic current should be employed.

None of these methods should be brought into frequent use; they are for emergencies only.

I cannot resist again calling your attention and consideration to several points which are to me of profound interest.

The development of the uterus by slow degrees through nine months of pregnancy does not seem very difficult of apprehension, but quite otherwise is its suddenly undertaking such great labors, like a giant developed in an hour, and then its change immediately afterward from a large sack containing gallons to a firm hard ball with a cavity holding but a few ounces.

Again, I have been accustomed to regard that condition of the post-partum uterus, which was alternately hard and soft, as a condition of danger; that the retractility, or *tonic* contractility of the organ was deficient, although the *active* contractility seemed good enough. I am inclined still to hold this view, and to think clots remaining in the uterus a twofold source of danger, although much may be said upon the other side to induce a conclusion that instead of being dangerous this is the most natural condition and one which should not be disturbed. I understand that Dr. Groesbeck of our own city holds this view. The whole subject will bear further study.

The tendency of to-day in the de livery of the placenta, seems to be to approach the methods of Mauriceau and Burton, at least so far as to deliver immediately. Several eminent gentlemen I am told recommend to introduce the hand for it, if not delivered in ten minutes. Burton's French editor (ed. 1751) said the plan was dangerous, because profuse hæmorrhage might follow,—that atony was thereby produced. His conclusion, however, seems doubtful. He says nothing of the danger of producing an acute inflammation, which most modern authors think liable to follow such a procedure. I have yet to learn of any case where metritis followed a simple introduction of the hand and withdrawal of the placenta. I ask, then, the question, how great is the danger of acute inflammation of the uterus, from the careful introduction of the hand into it immediately after the expulsion of the child? Is it any greater or indeed as great as by energetically following Crede's method? Either method I regard as suitable only for emergencies, but is not Burton's as harmless as Crede's?

